# Anti-bladder cancer effects of nuciferine, a bioactive compound extracted from *Nelumbo nucifera* leaves

**DOI:** 10.3389/fnut.2026.1881388

**Published:** 2026-10-20

**Authors:** Bin Qin, Hong Liang, Tongdong Kuang, Chaohe Wei, Meihui Qin, Jiayao Li, Huiling Lu, Chao Guo

**Affiliations:** 1 Department of Urology Surgery, Guigang City People's Hospital, The Eighth Affiliated Hospital of Guangxi Medical University, Guigang, China; 2 Department of Pharmacy, Guigang City People's Hospital, The Eighth Affiliated Hospital of Guangxi Medical University, Guigang, China; 3 Key Laboratory of Environmental Pollution and Integrative Omics, Education Department of Guangxi Zhuang Autonomous Region, Guilin Medical University, Guilin, China; 4 Sub-Center of Key Laboratory of Environmental Pollution and Integrative Omics (Education Department of Guangxi Zhuang Autonomous Region), Lingshan Hospital of Guilin Medical University, Lingshan, China

**Keywords:** bladder cancer, instrument analysis, nuciferine, potential target, tumor growth

## Abstract

Nuciferine, a bioactive compound found in *Nelumbo nucifera* leaves, demonstrates diverse pharmacological activities, including anticancer effects. However, to our knowledge, no previous study has examined the effects of nuciferine on bladder cancer (BC). In this study, nuciferine was first isolated from locally sourced *N. nucifera* leaves and characterized using instrumental analysis. Signal transducer and activator of transcription 3 (STAT3), a potential anti-BC target, was identified through molecular docking analysis. *In vivo* assays demonstrated that nuciferine inhibited tumor growth in BC cell-based tumor-bearing mice. Nuciferine-treated mice showed an increase in terminal deoxynucleotidyl transferase dUTP nick-end labeling (TUNEL)-positive cells and a decrease in Ki67-labeled cells. In addition, B-cell lymphoma 2 (Bcl-2) and STAT3 protein levels were reduced, whereas that of Bcl-2-associated X (Bax) was elevated following nuciferine treatment in mice. In conclusion, our findings suggest that nuciferine, a bioactive compound extracted from *N. nucifera* leaves, represents a potential candidate for preclinical treatment of BC.

## 1 Introduction

Globally, bladder cancer (BC) is one of the most refractory malignant tumors, characterized by increasing incidence and poor prognosis (1). BC incidence and mortality rates are highest in developed regions, such as North America and Southern Europe (2). According to epidemiological statistics, new BC cases in China account for approximately one-sixth of all global cases, ranking it among the major malignant tumors in the country (3). Reports suggest that certain high-risk factors, including cigarette smoking, occupational exposure, industrialized food products, and dysregulated microbiome, may contribute to BC development (4). Genetic mutations, particularly in chromatin-modifying genes, constitute the molecular basis of BC initiation and progression. Key examples include alterations in phosphatidylinositol 3-kinase catalytic subunit alpha (PIK3CA), fibroblast growth factor receptor 3 (FGFR3), and lysine-specific methyltransferase 2D (KMT2D) (5). Currently, the medical diagnosis of BC is based on analysis of urinary biomarkers, standard histopathology, multi-parametric magnetic resonance imaging (MRI), and cystoscopy (6). Chemotherapy for BC commonly involves cisplatin, methotrexate, doxorubicin, and vinblastine. However, these therapies are frequently associated with adverse effects (7, 8). Therefore, there is an urgent need to explore and identify compounds with lower toxicity or higher efficacy, especially those derived from natural products.

Previous studies have reported the use of traditional Chinese medicine (TCM) and certain bioactive extracts in anticancer therapy that mediate their effects through various pharmacological mechanisms, such as suppression of cell proliferation, angiogenesis, and metastasis, induction of cell death and apoptosis, and activation of immunopotentiation (9). *Nelumbo nucifera*, widely distributed across China and rich in isoquinoline-type alkaloids, has long been used as a traditional medicinal plant (10). Nuciferine, a functional alkaloid extracted from the leaves of *N. nucifera*, has demonstrated promising biological activities, including reduction of hyperlipidemia and blood pressure, amelioration of metabolic insufficiency, and alleviation of oxidative stress (11). As such, nuciferine may serve as a candidate for prophylaxis and treatment of inflammation-associated disorders, including human cancers (12). In a prospective pilot study, nuciferine was used for the management of nocturia, urgency, and dysuria in patients (13). Notably, nuciferine was found to inhibit the proliferation and progression of oral squamous cell carcinoma partially through STAT3 inactivation both *in vitro* and *in vivo* (14). Therefore, in the present study, we aimed to investigate the pharmacological activity of nuciferine and to identify its potential molecular targets in BC. *N. nucifera* leaves (obtained from the Guangxi Zhuang Autonomous Region) were used for the isolation and identification of nuciferine through pharmaceutical chemistry analyses. Molecular docking simulation analysis was used to screen and characterize signal transducer and activator of transcription 3 (STAT3) as a potential anti-BC target of nuciferine. In addition, the anti-BC pharmacological activity of nuciferine was demonstrated *in vivo* in a tumor-bearing mouse model, and the endogenous expression of STAT3 in nuciferine-treated tumors was analyzed.

## 2 Materials and methods

### 2.1 Extraction and identification of nuciferine

*N. nucifera* leaves (0.5 kg) were obtained from Guigang City (Guangxi Zhuang Autonomous Region). A voucher specimen (No. 2026NNG001) was deposited in the Guangxi Key Laboratory of Diabetic Systems Medicine, Guilin Medical University. To enable targeted tracking of the bioactive compound nuciferine in the leaves of *N. nucifera*, a comprehensive strategy integrating thin-layer chromatography (TLC), high-performance liquid chromatography (HPLC), and liquid chromatography-mass spectrometry (LC–MS) was implemented for its isolation and identification. The leaves of *N. nucifera* were finely powdered and extracted with 80% ethanol by refluxing. The ethanol distillate was extracted with ethyl acetate and then fractionated by silica gel column chromatography using a CH_2_Cl_2_/MeOH gradient (100:1, 50:1, *v/v*), producing three fractions (Fr.1–3). Fraction 3 (110.0 mg) was purified by preparative HPLC on a C_18_ column with a CH_3_OH/H_2_O mobile phase (82:18, *v/v*), yielding nuciferine (5.0 mg).

### 2.2 Molecular docking assay

Molecular docking simulation analysis was performed to investigate and characterize STAT3 as a target protein *in silico*. The bioactive compound “nuciferine” was retrieved from the PubChem database, and its molecular structure was obtained. The three-dimensional structure of STAT3 was acquired from the Protein Data Bank (PDB) (https://www.rcsb.org/). Subsequently, dehydration, hydrogenation, and charge assignment were performed via AutoDock software, and the modified STAT3 and nuciferine structures were exported as PDBQT files. Docking progress was screened using the AutoDock Vina software, and the output data were imported into PyMOL software for further validation and visualization of the binding.

### 2.3 Animal experimental design

Mature male C57BL/6 mice (6 weeks old) were purchased from Hubei Bainte Biotechnology Co., Ltd. (Wuhan, China). After one week of adaptation, cell-based xenograft mouse models were established by aseptically injecting stably cultured mouse BC MB49 cells (1 × 10^6^/mouse) into the right axilla of mice. After allowing the tumors to grow for 7 days, nuciferine (40 mg/kg body weight; treatment group, *n* = 6 mice) or solvent (control group, *n* = 6 mice) was administered via intragastric gavage once daily for 10 days. This animal study was approved by the Experimental Animal Ethics Committee of the Guangxi Medical University (No. 202509025). At the end of the treatment period, all mice were euthanized, and the solid tumors were collected. The tumor weight was recorded, and the tumor tissue was then fixed with 4% paraformaldehyde prior to further analyses.

### 2.4 Immunofluorescence staining

Tumor samples were processed into 5 μm paraffin-embedded sections. Following dewaxing, the sections were permeabilized with Triton X-100 and blocked with 5% fetal calf serum (v/v). The sections were then incubated with primary antibodies against Bcl-2, Bax, or STAT3 (1:1000, Servicebio, Wuhan, China) overnight at 4 °C. After washing, the samples were incubated with a secondary antibody (coupled with red fluorescence, Servicebio, Wuhan, China) at 37 °C for 1 h. The nuclei were stained with 4′,6-diamidino-2-phenylindole (DAPI, Servicebio). Following washes, the fluorescently labeled cells were imaged using a fluorescence microscope (Nikon, Japan), and the data were analyzed.

### 2.5 Statistical analyses

Raw data were analyzed using the GraphPad Prism software (GraphPad, USA). The data are presented as mean ± standard deviation. Statistical differences were analyzed using the Student’s *t*-test, followed by one-way analysis of variance. Statistical significance was set at *p* < 0.05.

## 3 Results

### 3.1 Pharmaceutical analysis of nuciferine

Using reference standards, phytochemical studies of *N. nucifera* leaves led to the isolation of a nuciferine-related target compound. We first sought to demonstrate the isolation and structural elucidation of the bioactive compound. To enable tracking of nuciferine in the leaves, LC–MS analysis was performed, and ongoing phytochemical procedures were used for the isolation and characterization of the target compound. The bioactive compound was obtained as white powder with the molecular formula C_19_H_21_NO_2_, confirmed by high-resolution electrospray ionization mass spectrometry (HRESIMS) at *m/z* 296.1640 [M + H]^+^. ^1^H-NMR (400 MHz, DMSO-*d*_6_): *δ* 8.17 (1H, dd, *J* = 7.8, 1.3 Hz, H-11), 7.32 (1H, d, *J* = 6.1 Hz, H-10), 7.29 (1H, d, *J* = 7.7 Hz, H-9), 7.23 (1H, dd, *J* = 7.3, 1.4 Hz, H-8), 6.77 (1H, s, H-3), 2.64–3.17 (7H, m, H-4, 5, 6a, 7), 3.80 (3H, s, 1-OMe), 3.57 (3H, s, 2-OMe). ^13^C-NMR (101 MHz, DMSO-*d*_6_): *δ* 144.5 (s, C-1), 151.6 (s, C-2), 111.9 (d, C-3), 28.7 (t, C-4), 152.6 (t, C-5), 62.0 (d, C-6a), 34.4 (t, C-7), 136.5 (s, C-7a), 128.1 (d, C-8), 127.6 (d, C-9), 126.8 (d, C-10), 127.6 (d, C-11), 131.6 (s, C-11a), 125.9 (s, C-11b), 128.9 (s, C-11c), 59.7 (q, 1-OMe), 55.7 (q, 2-OMe). Based on these data (Figure 1), the target compound was identified as nuciferine.

**Figure 1:**
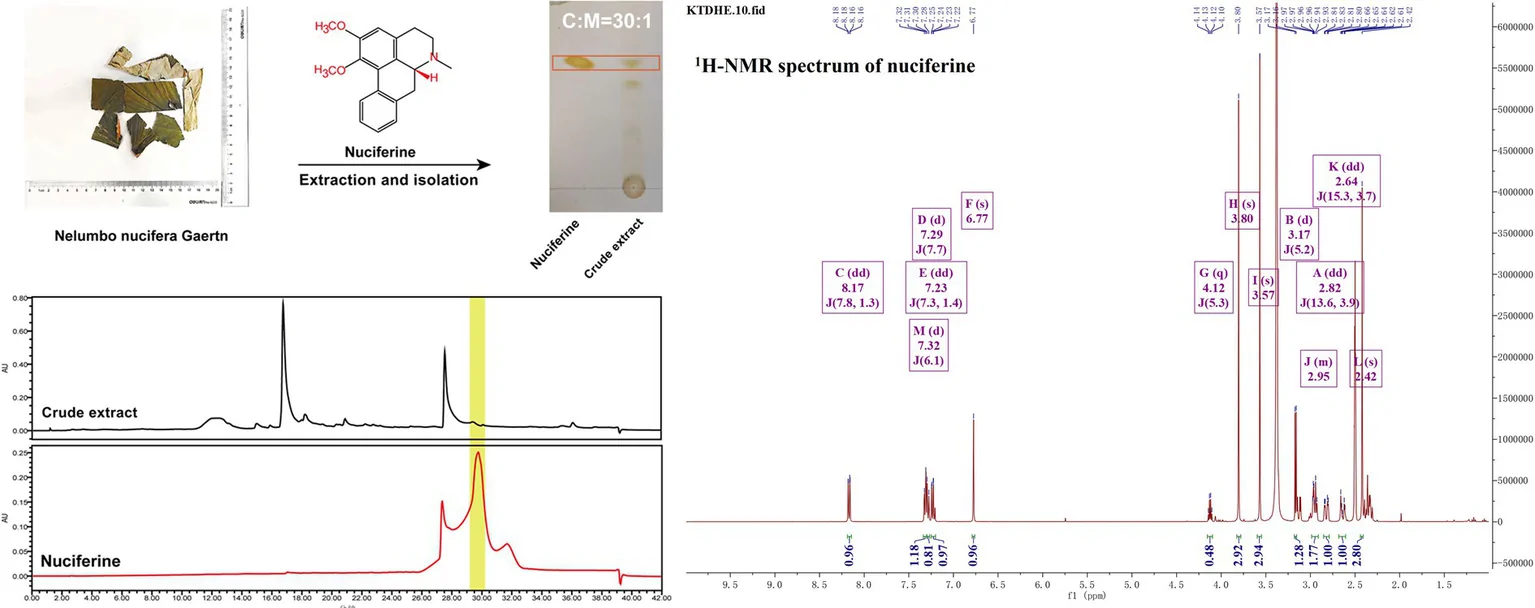
Flowchart showing nuciferine isolation from the leaves of *Nelumbo nucifera* and instrumental analysis-based identification.

### 3.2 Molecular docking data

To determine whether nuciferine directly interacts with STAT3 in BC, a molecular docking simulation assay was conducted. Nuciferine demonstrated a free binding energy of −6.2 kcal/mol with the STAT3 protein, forming hydrogen bonds with the amino acid residues GLN-644 and TYR-657 in the STAT3 protein (PDB ID: 6NJS; Figure 2), indicating stable binding affinity and emphasizing the role of hydrogen bonds in stabilizing the nuciferine-STAT3 complex.

**Figure 2:**
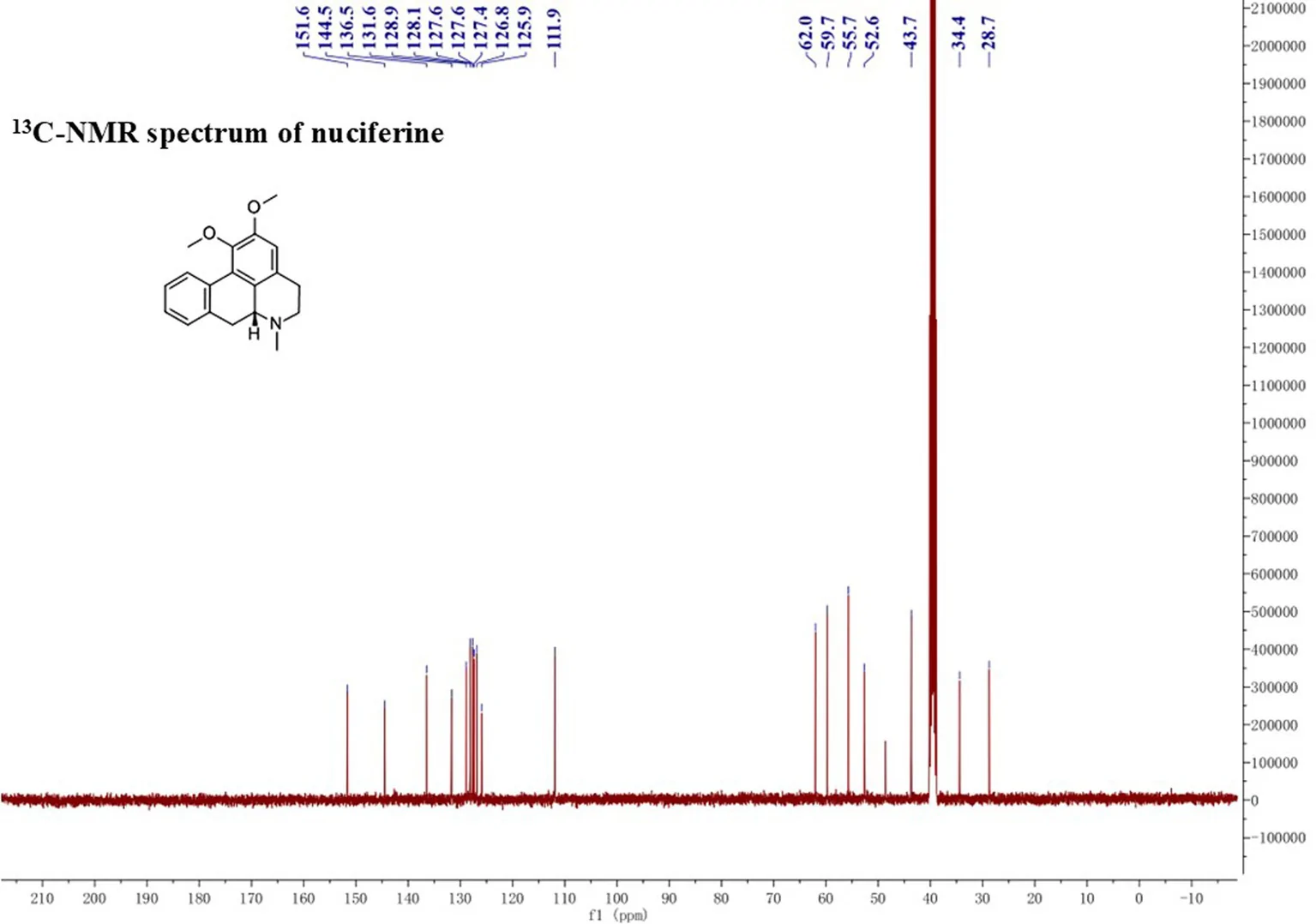
^13^C-NMR spectrum of nuciferine.

### 3.3 Nuciferine suppresses BC cell growth *in vivo*

To examine the pharmacological effects of nuciferine on BC cell growth in tumor-bearing mice, the expression of apoptosis-related regulators was evaluated following nuciferine treatment (see Figures 3, 4). Nuciferine treatment significantly reduced tumor growth in mice when compared with untreated controls (*p* < 0.05) (Figure 5A). Immunofluorescence staining revealed that TUNEL-labeled and Bax-positive cells were increased, whereas Bcl2-positive cells were decreased in nuciferine-treated mice when compared with the untreated controls (*p* < 0.05) (Figure 5B). As further validation of the molecular docking data, immunostaining showed that STAT3-labeled cells were decreased in nuciferine-treated mice compared with the controls (*p* < 0.05) (Figure 5C). These results suggest that the anti-BC activity of nuciferine may be partially mediated via inhibition of the apoptosis-related STAT3 signaling pathway.

**Figure 3:**
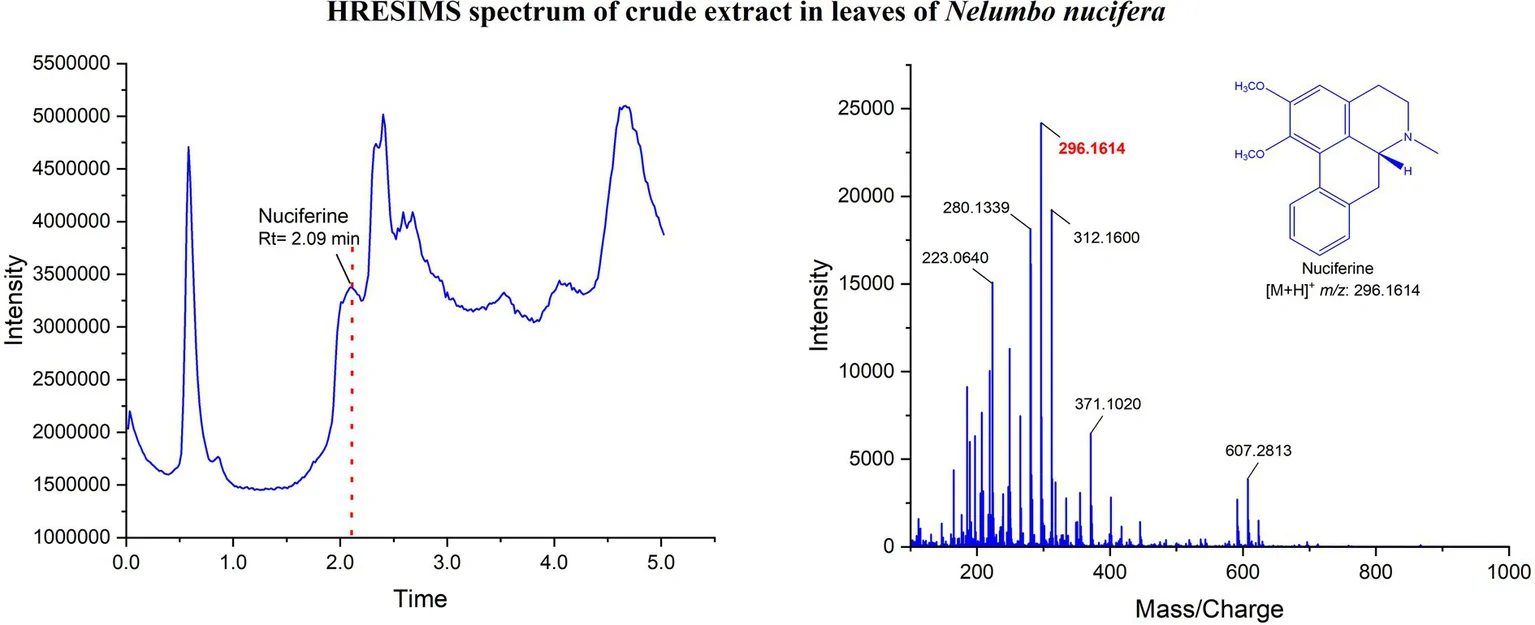
HRESIMS spectrum of crude extract in Nelumbo nucifera leaves.

**Figure 4:**
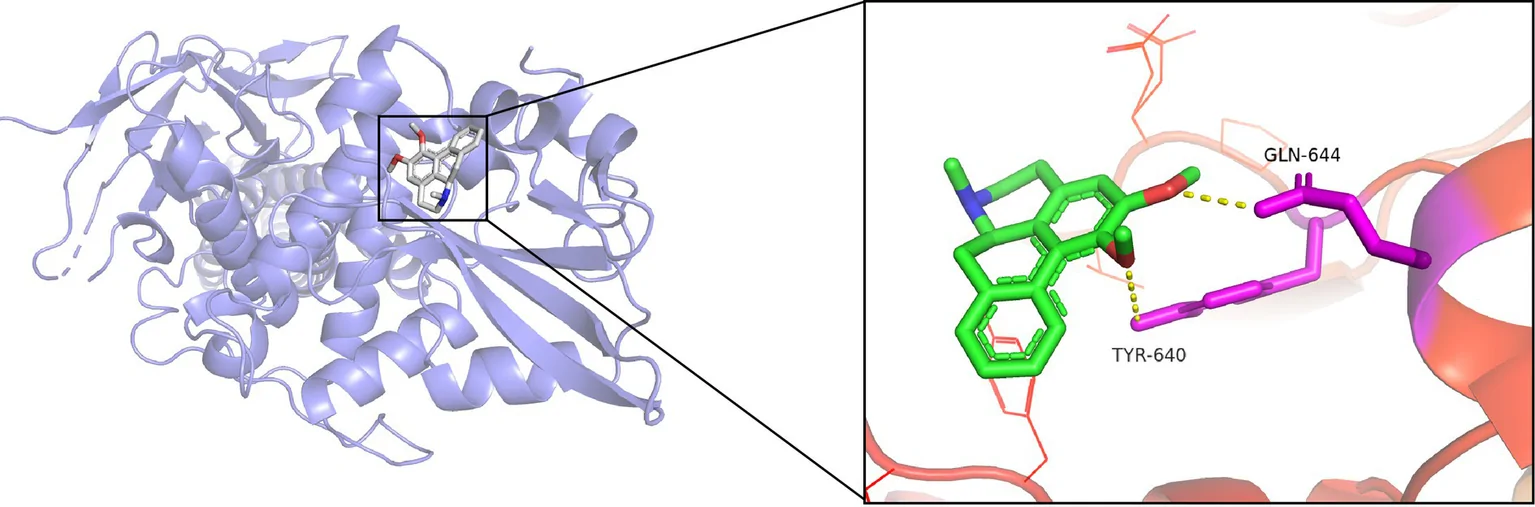
Spatial docking modes of nuciferine with signal transducer and activator of transcription 3 (STAT3) visualized in three-dimensional representation..

**Figure 5:**
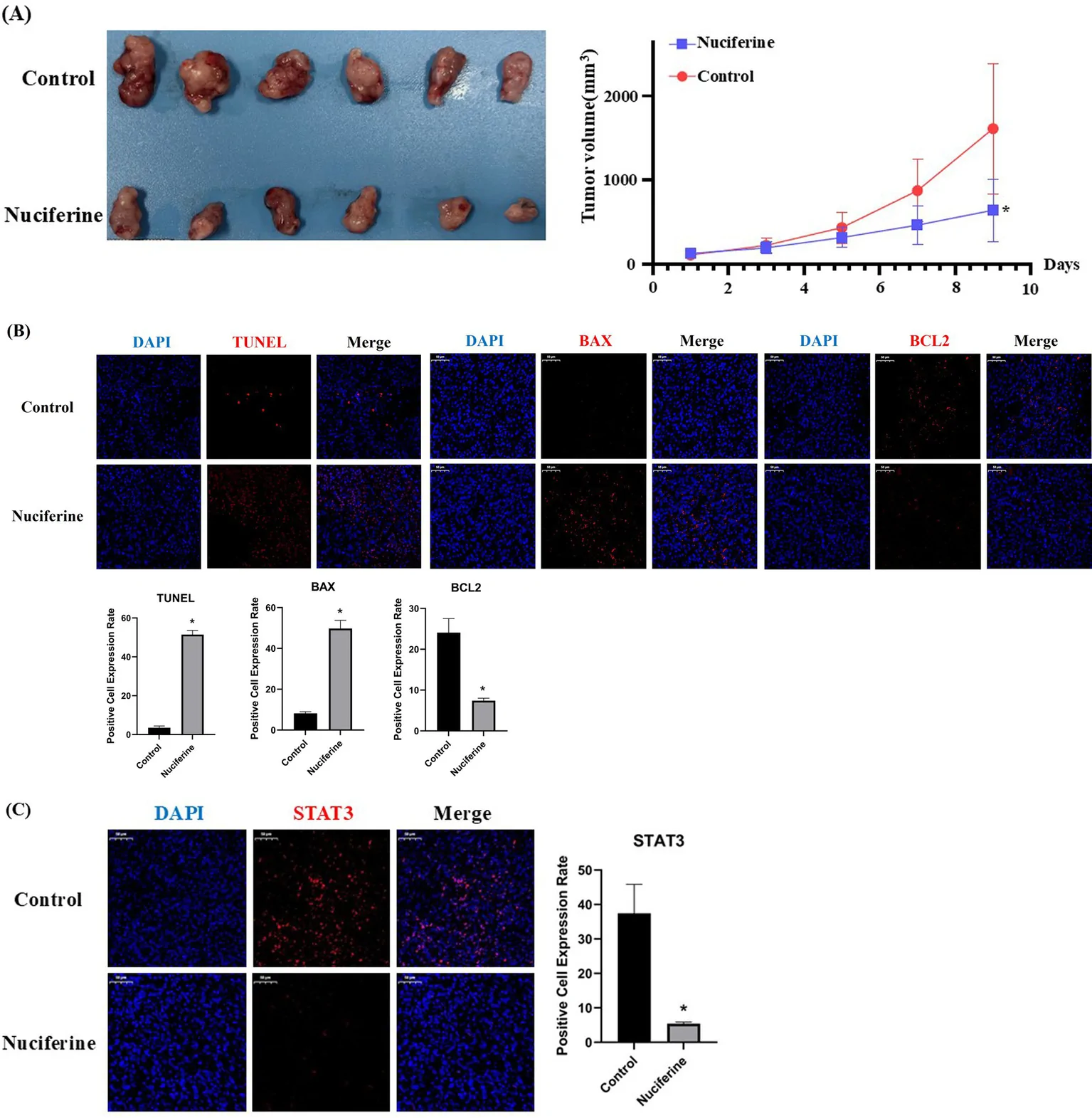
Nuciferine suppresses bladder cancer (BC) tumor growth *in vivo* through regulation of signal transducer and activator of transcription 3 (STAT3)-related apoptosis. **(A)** Nuciferine inhibits BC tumor growth in mice (*p < 0.05; *n* = 6). **(B)** Nuciferine induces apoptosis and apoptosis-related regulators in tumor samples. **(C)** Nuciferine inactivates STAT3 in tumor samples.

## 4 Discussion

For centuries, the leaves of *N. nucifera* have been widely used in China for medicinal purposes (15). Nuciferine, a biofunctional component extracted from *N. nucifera*, demonstrates promising activities against cancer (16). However, the anticancer effects of nuciferine against BC have not yet been investigated. Therefore, we first isolated the nuciferine compound from *N. nucifera* leaves sourced from Guangxi, followed by instrumental analysis and ingredient identification. Preliminary data indicated that nuciferine is abundant in *N. nucifera* leaf extracts and may represent one of the main bioactive compounds in the leaves. The anticarcinogenic action and molecular target of nuciferine in BC were assessed using *in vivo* and *in silico* experiments. Molecular docking simulation has emerged as a key computational tool for screening potential targets in the drug discovery process, including naturally occurring compounds (17). Based on prior research and molecular docking analysis, STAT3 was identified as a potential pharmacological target of nuciferine in BC. The STAT3 pathway plays an important role in regulating inflammatory reactions, tumorigenesis, and other pathological processes. As a transcriptional factor, STAT3 may serve as a potential therapeutic target in diverse diseases (18). STAT3 is implicated in several cancer hallmarks, including stemness, proliferation, angiogenesis, transformation, and drug tolerance. Thus, STAT3 represents a promising target for cancer treatment (19). STAT3 has key roles in carcinogenic processes, such as apoptosis, cell cycle, and uncontrolled growth, indicating that STAT3 may contribute to cancer development via apoptosis and associated pathways (20). In the present study, nuciferine treatment of tumor-bearing mice resulted in reduced tumor growth, induction of apoptosis (elevated TUNEL and Bax expression and reduced Bcl-2 expression), and suppression of STAT3 activity. TUNEL assays are extensively used to assess apoptotic cell death, especially in oncology research (21). Bax functions as a pivotal executioner in mitochondria-regulated cell death. Bax dysregulation induces abnormal cell death via disruption of the mitochondrial membrane (22). Numerous anticancer drugs, including small-molecule agents, exert their effects through indirect activation of Bax, which represents a promising pharmacological target for anticancer drug discovery (23). Bcl2 plays a critical role in regulating apoptotic cell death. Abnormal activation of Bcl2 contributes to the initiation and progression of malignant tumors (24). Bax/Bcl-2 may serve as a key parameter for assessing anti-BC targets based on apoptosis regulation (25). On the basis of current reference assays, these data demonstrate that nuciferine suppressed BC tumor growth via inactivation of apoptosis-related STAT3 expression. Based on *in silico* analysis, STAT3 was identified as a pharmacological target of nuciferine in BC. Further *in vivo* validation experiments revealed that nuciferine exerts anti-BC effects by reducing neoplasm-promoting STAT3 activity.

However, this study has some limitations. The experimental setup was constrained by small comparative groups. It also lacked detailed cell culture experiments, and the pharmacological mechanisms remain unexplored. The correlation between nuciferine treatment and the STAT3 signaling pathway should be established further using *in vitro* and *in vivo* approaches. Future investigations should also examine the functional loss of STAT3 to better understand the mechanisms underlying the anticancer effects of nuciferine in BC.

## 5 Conclusion

Taken together, current experimental findings demonstrate that nuciferine may contribute to suppression of BC tumor growth by inducing apoptosis. Preliminary mechanistic data suggest that the inhibitory effects of nuciferine in BC may be mediated through direct inactivation of STAT3 expression. Thus, nuciferine represents a promising bioactive compound for the management of BC.

## Data Availability

The raw data supporting the conclusions of this article will be made available by the authors, without undue reservation.
